# Supplementary material for: Splice-Junction-Based Mapping of Alternative Isoforms in the Human Proteome
Source: Cell Rep. Author manuscript; Available in PMC 2020 Jan 15. (PMC6961840; doi:10.1016/j.celrep.2019.11.026)
Supplement: 2 [file NIHMS1546469-supplement-2.zip › DF1/PXD010154/Spleen/Spleen_19_MRNIP_EAVLFAGESYK.pdf]

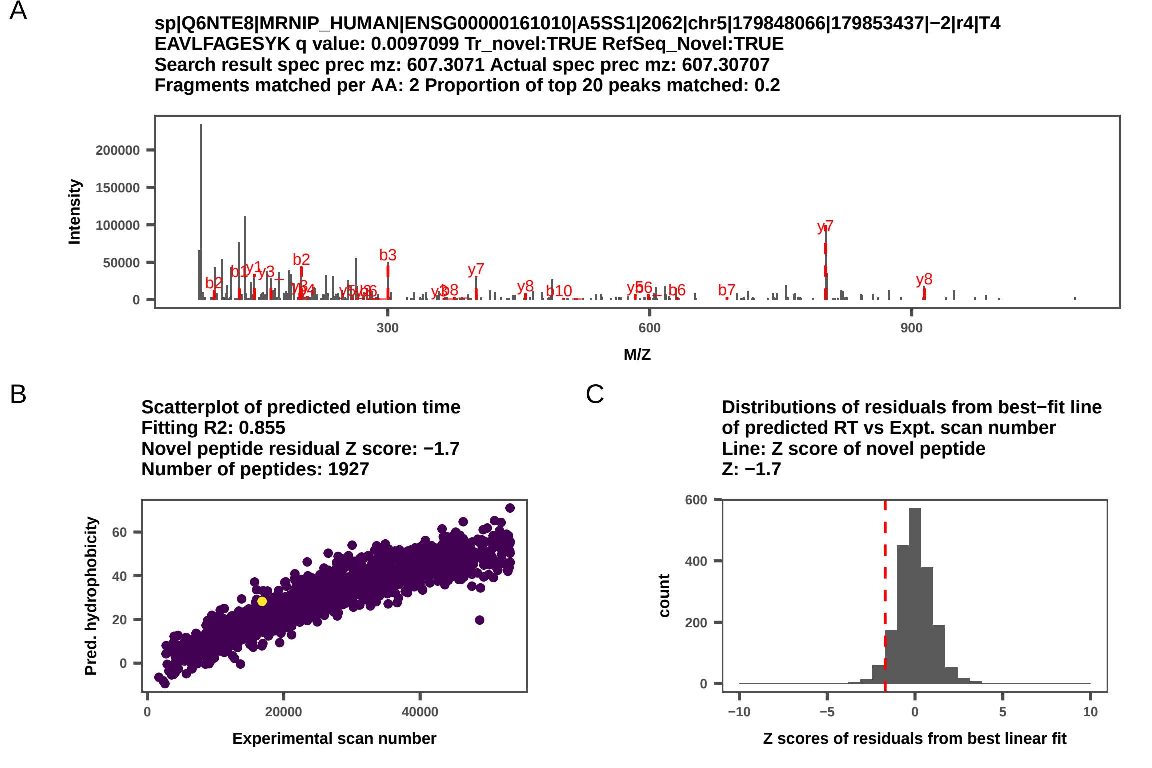
A
sp|Q6NTE8|MRNIP_HUMAN|ENSG00000161010|A5SS1|2062|chr5|179848066|179853437|−2|r4|T4
EAVLFAGESYK q value: 0.0097099 Tr_novel:TRUE RefSeq_Novel:TRUE
Search result spec prec mz: 607.3071 Actual spec prec mz: 607.30707
Fragments matched per AA: 2 Proportion of top 20 peaks matched: 0.2
Intensity
200000
150000
100000
50000
0
300
600
900
M/Z
B
Scatterplot of predicted elution time
Fitting R2: 0.855
Novel peptide residual Z score: −1.7
Number of peptides: 1927
Pred. hydrophobicity
60
40
20
0
0
20000
40000
Experimental scan number
C
Distributions of residuals from best−fit line
of predicted RT vs Expt. scan number
Line: Z score of novel peptide
Z: −1.7
count
600
400
200
0
−10
−5
0
5
10
Z scores of residuals from best linear fit
